# A Children's Hospital in Difficulties

**Published:** 1917-09-15

**Authors:** E. J. Fattorini

**Affiliations:** Hon. Secretary. Bradford Children's Hospital


### A Children's Hospital in Difficulties.

*To the Editor of* The Hospital.

Sir,—In reference to the editorial in your issue of August 11, we should like to point out that it has no justification whatever. As regards the first sentence we know of no complaints except those coming from ourselves in regard to parents who bring in their children and make a declaration that there is no infectious disease in their house, knowing perfectly well that they have left a case of measles at home. As to "the fact being admitted" that we have been "placed in a disagreeable position," (1) we know of no such position, (2) we know of no one who has admitted the fact.

The last paragraph—namely, "The matter has become sufficiently serious to call for immediate investigation, as, in the interest alike of the children and the institution, the Bradford Children's Hospital must clear itself of the

(Continued on page 497.)

### THE EDITOR'S LETTER-BOX (*continued from p.* 491).

### A Children's Hospital in Difficulties—(*continued*).

prejudice which, unfortunately, has been created against it in the minds of parents in this respect "—is more misleading, as the following statistics will show :—

During May we had four cases : one of cerebro-spinal meningitis and three of measles. During June we had no cases of infection. During July we had four cases of measles. During August we had two cases of measles and one of cerebro-spinal meningitis.

Considering that the city has been swept by an epidemic of measles of extraordinary severity during this time, and that we have not had a single complaint, we would ask you to kindly publish this letter so as to make it clear that the complaint, if any, comes from the hospital authorities and not from outside.

(Signed on behalf of the Board of Management),

E. J. FATTORINI, Hon. Secretary.

Bradford Children's Hospital, August 31, 1917.

[While it is satisfactory to learn from Mr. E. J. Fattorini that the Bradford Children's Hospital has been so comparatively little invaded by infectious disease considering the epidemic of measles which has "swept the city," it is surprising to hear that the hospital has not "had a single complaint." We need go no further than a speech delivered by Mr. Spence at the meeting of the Bradford Hospital Fund on July 26. In the *Yorkshire Observer* of the following day Mr. Spence was reported as saying that "there had been renewed complaints about the children who had been sent to the institution developing infectious disease almost immediately after admission. It was pretty clear that they had been infected before admission. Great inconvenience and loss were entailed in consequence, and something would have to be done to stop the practice." Here is abundant suggestion of complaint, and ample evidence of "a disagreeable position." But the latter, indeed, is, alas ! only too plain from Mr. Fattorini's own statistics.—ED. THE HOSPITAL.]

### The British Nursing Profession : The Two Voices.

*To the Editor of* THE HOSPITAL.

